# Carbachol protects the intestinal barrier in severe acute pancreatitis by regulating Cdc42/F-actin cytoskeleton

**DOI:** 10.3892/etm.2020.8985

**Published:** 2020-07-10

**Authors:** Hanlin Wang, Yingjian Jiang, Hongbo Li, Jiang Wang, Chang Li, Dianliang Zhang

**Affiliations:** Center of Colon and Rectum, Qingdao Municipal Hospital, Qingdao University, Qingdao, Shandong 266011, P.R. China

**Keywords:** carbachol, pancreatitis, tight junction, bacterial translocation

## Abstract

The present study aimed to investigate the effect of carbachol on the intestinal tight-junction barrier in a rat model of severe acute pancreatitis (SAP) without aggravating pancreatic injury, and to determine whether cell division cycle 42 (Cdc42)/F-actin could have a regulatory role. Rats were separated into a sham-operation (SO) group (n=10), SO + carbachol group (n=10), SAP group (n=60) and SAP + carbachol group (n=60). Sodium taurocholate (5%) was retrogradely injected into the biliopancreatic duct of rats to induce SAP. Subsequently, 16S rRNA sequencing was used to detect bacterial translocation (BT) in the gut of surviving animals. Hematoxylin and eosin staining was used to detect morphological changes in the pancreas and intestine. The expression of F-actin and tight junction proteins was analyzed by western blotting and immunofluorescence, and Cdc42 expression was analyzed by immunohistochemistry and western blotting. The results demonstrated that the intestinal injury in SO and SO + carbachol groups was lower than that in the SAP + carbachol group (P<0.05); however, the intestinal injury was similar in the SO and SO + carbachol groups (P>0.05), and was significantly more severe in the SAP group compared with the SAP + carbachol group (P<0.05). Similarly, pancreatic injury in the SAP and SAP + carbachol groups was significantly higher compared with the SO and SO + carbachol groups (P<0.05); however, pancreatic injury was similar in the SAP and SAP + carbachol groups (P>0.05), and in the SO and SO + carbachol groups (P>0.05). Furthermore, the mortality rate and BT in the SAP group were significantly higher compared with the SAP + carbachol group (mortality rate, 50% vs. 30%, P<0.05; BT, 60% vs. 33.3%, P<0.05). In addition, the expression of Cdc42, F-actin and claudin-2 was significantly higher in the SAP and SAP + carbachol groups compared with the SO and SO + carbachol groups (P<0.05), and the expression of occludin and zonula occludens-1 were significantly higher in the SO and SO + carbachol groups compared with the SAP and SAP + carbachol groups (P<0.05). In conclusion, these findings demonstrated that carbachol may protect the intestinal barrier in the SAP rat model without aggravating pancreatic injury via regulation of Cdc42/F-actin expression.

## Introduction

Severe acute pancreatitis (SAP) is an acute disease characterized by a complex pathogenesis that causes rapid failure of numerous organs (including renal, respiratory and/or cardiovascular organs) (1). The global mortality rate of patients with SAP ranges from 13-35% (2,3) and can reach 70% in case of pancreatic infection (4-9). However, surgical treatment of SAP is also associated with high complication (34-95%) and mortality (11-39%) rates (8,10). The risk of mortality from multiple organ dysfunction and sepsis following surgery remains high. Patients with SAP mainly succumb to infection, mostly secondary pancreatic infection and infection-related organ failure in the later stages (11). The intestinal tract is the central organ location of the SAP-induced systemic inflammatory response syndrome (SIRS). It has been demonstrated that in SAP, the intestinal mucosa is damaged and intestinal permeability is increased (12,13), and bacterial translocation (BT) consists of the entry of intestinal bacteria and endotoxins into the circulatory and lymphatic systems and distant organs, passing through the intestinal barrier (14,15). BT from the gastrointestinal tract is considered as the main cause of sepsis and systemic infection in patients with SAP (16,17). Previous studies have reported that in SAP, the intestinal tight junction (TJ) barrier is destroyed and intestinal mucosal permeability is increased, thereby facilitating BT (18,19), indicating that intestinal epithelial TJs serve a crucial role in the regulation of intestinal mucosal barrier function (20,21).

TJs mainly comprise intercellular transmembrane proteins, including claudin and occludin, which are linked to different peripheral membrane proteins located inside the plasma membrane, mainly to zonula occludens-1 (ZO-1) (22). TJs form a physical barrier between mucosal epithelial cells. The F-actin cytoskeleton is involved in numerous important cellular processes, including intercellular junctions, cell morphology, cell movement and signal transduction, which are mediated by the interaction between F-actin and the cell membrane (23). Cell division cycle 42 (Cdc42) is a widely expressed Rho family GTP-binding protein that regulates actin skeleton assembly and rearrangement, thereby affecting cell morphology, migration and endocytosis (24).

Carbachol is a cholinergic agonist that promotes gastrointestinal motility, increases glandular secretion and protects the intestinal barrier (25-27). A previous study demonstrated that carbachol increases intestinal transmembrane electrical resistance and protects the intestinal barrier in a rat model of inflammatory bowel disease (IBD) (26). In addition, carbochol can reduce intestinal barrier permeability and TJ damage induced by intraperitoneal injection of lipopolysaccharide (LPS) (27). A previous meta-analysis demonstrated that octreotide, a synthetic somatostatin analogue used to treat moderate to severe acute pancreatitis, has no significant benefits on the clinical outcomes of patients (28). Furthermore, a long-term clinical study reported that the clinical benefits of somatostatin or octreotide in patients with SAP are not primarily achieved by inhibiting pancreatic secretion, since a large number of pancreatic acinar lesions and necrosis in patients with SAP result in insufficient residual acinar exocrine secretion (29). It is hypothesized that it may be possible to protect the SAP intestinal barrier by intraperitoneal injection of carbachol without aggravating pancreatic injury.

Numerous studies reported that carbachol can protect the intestinal barrier (25-27); however, its role in the intestinal barrier in patients with SAP remains unknown. The present study aimed to investigate the effect of carbachol on the intestinal TJ barrier in a rat model of SAP. The results suggested that carbachol may prevent intestinal barrier injury in SAP rats by regulating the Cdc42-F-actin cytoskeleton, without aggravating pancreatic injury.

## Materials and methods

### 

#### Animal model

A total of 140 7-week old Wistar rats (healthy males; 230-260 g; Animal Center of Qingdao University) were acclimated to the laboratory over 3 weeks and were then randomly assigned into the sham operation (SO) group (n=10), SO + carbachol group (n=10), SAP group (n=60), and SAP + carbachol group (n=60). All animals were housed at standard temperatures (25±2˚C) and at a relative humidity (50-70%) under a 12 h light/dark cycle with free access to food and water. All experimental protocols were performed according to the National Institutes of Health Laboratory Animal Guidelines and were approved by the Institutional Animal Care and Use Committee of Qingdao University.

The SAP rat model was constructed as previously described (30). Briefly, the rats fasted for 12 h, and were then weighed and anesthetized using an intraperitoneal injection of pentobarbital sodium (3%; 50 mg/kg). During laparotomy, the biliary pancreatic duct was clipped near the hepatic portal and duodenal intubation was performed with a catheter. Sodium taurocholate (30) (5%; 1 ml/kg) was slowly injected into the biliopancreatic duct to induce SAP. For the SAP + carbachol group, carbachol (50 µg/kg) was injected intraperitoneally 12 h following SAP induction. For the SAP group, rats were intraperitoneally injected with the same volume of sterile saline 12 h following SAP induction. In the SO group, the same amount of sterile saline was injected into the biliopancreatic duct and abdominal cavity of rats. In the SO + carbachol group, carbachol (50 µg/kg) was injected intraperitoneally.

All surviving rats were anesthetized 24 h following SAP induction and treatments. Blood samples were collected from the inferior vena cava and were divided into two microtubules. One microtubule was centrifuged at 4˚C and 3,000 x g for 5 min. The supernatant was collected and kept in an Eppendorf tube at -20˚C for analysis of serum lipase and amylase. The second microtubule containing EDTA was stored at -20˚C for bacterial DNA analysis. Finally, the ileum and pancreatic tissue were isolated and the ileum was divided into two sections. The first section was stored at -80˚C for western blot analysis, and the second section, along with pancreatic tissue, was fixed in 4% paraformaldehyde at room temperature for 48 h.

#### Bacterial detection by 16S rRNA sequencing

BT was assessed in the present study by detecting the presence of bacteria in rat blood. Bacterial DNA was isolated from bacterial colonies using a Rapid Bacterial Genomic DNA Isolation kit (cat. no. B518225; Sangon Biotech Co., Ltd.) according to the manufacturer's protocol, and isolated DNA was, as a template, used to amplify the hypervariable regions (V6-V8) of the 16S rRNA gene. R1521-1539 (5'-AGGAGGTGATCCAACCGCA-3') and F1169-1187-GC (5'-GC-clamp-AACTGGAGGAAGGTGGGGA-3'; Sangon Biotech Co., Ltd.) were used as the universal primers. Negative and positive controls were assessed twice to avoid false-positive results.

PCR was performed using a touchdown thermocycling program after adding 100 ng DNA to the reactant. The following thermocycling conditions were used: Initial denaturation for 5 min at 95˚C followed by denaturation for 25 cycles for 30 sec at 95˚C, annealing for 30 sec at 55˚C and final extension for 5 min at 72˚C. PCR products were visualized on 2% agarose gel. DNA extracted from *E. coli* served as a positive control and ddH2O was used as a negative control. The final products were analyzed using a Roche GS FLX 454 Sequencer (Roche Diagnostics, Inc.). By using advanced BLAST (https://blast.ncbi.nlm.nih.gov/Blast.cgi) searches, the results of 16S rRNA sequence matched with those from GenBank (http://www.ncbi.nlm.nih.gov/genbank) and Ribosomal Database Project (http://rdp.cme.msu.edu) from National Center for Biotechnology Information.

#### Serum lipase and amylase

Serum lipase and amylase were detected with an Olympus AU600 automatic biochemical analyzer (Olympus Corporation), according to the manufacturer's instructions.

#### Histopathological score

Intestinal and pancreatic samples fixed in polyformaldehyde were embedded in paraffin. Sections (5-µm thick) were stained with hematoxylin and eosin at room temperature for 5 min and morphological changes were observed using an optical microscope (magnification, x200). The degree of pancreatic injury was assessed as previously described (31). The score ranged between 0 and 16 according to the degree of edema, acinar necrosis, hemorrhage and fat necrosis, and inflammation. Histological grading of small intestinal injury was evaluated as previously described (32). The grade ranged between 0 and 5 according to the degree of damage to the mucosal villi, subepithelial space, lamina propria, dilated capillaries, lifting epithelial layer and denuded tips of mucosal villi.

#### Immunofluorescence assay of intestinal F-actin and TJs

Immunostaining was performed as previously described (33), with samples that were previously fixed in PFA. Paraffin-embedded sections (5 µm) were dried at 37˚C for 15 min and boiled in 2 mM EDTA acid solution for 10 min. Non-specific binding sites were blocked with 1% bovine serum albumin (Roche Applied Science) and 5% (v/v) normal goat serum (Gibco; Thermo Fisher Scientific, Inc.) diluted in PBS at room temperature for 30 min. Sections were incubated overnight at 4˚C with the following primary antibodies: Rabbit anti-claudin-2 (1:500; Zymed; Thermo Fisher Scientific, Inc.; cat. no. 516100), rabbit anti-ZO-1 (1:500; Abcam; cat. no. ab96587) and rabbit anti-occludin (1:500; Zymed; Thermo Fisher Scientific, Inc.; cat. no. 711500). Sections were washed with PBS and were incubated with phalloidin-iFluor 594 (1:500; Abcam; cat. no. ab176757) and Alexa Fluor goat anti-rabbit IgG (1:500; 488 wavelength; Abcam; cat. no. ab150077) for 30 min at room temperature. Nuclei were stained with DAPI for 5 min. Images were captured using the IX71 fluorescence inverted microscope (Olympus Corporation). Fluorescence intensity was analyzed using Image J software (version 1.46; National Institutes of Health).

#### Immunohistochemistry (IHC)

IHC staining was performed for Cdc42 as previously described (34) by using the primary antibody rabbit anti-Cdc42 (1:500; Abcam; cat. no. ab187643) and the horseradish peroxidase-conjugated secondary antibody goat anti-rabbit IgG H&L (1:1,000; Abcam; cat. no. ab150080). Images were visualized with a fluorescent microscope (magnification, x200; DM6000B; Leica Microsystems GmbH) (34). IHC staining was scored as previously described (35) and following the German ImmunoReactive score system (36) according to the percentage of positively stained cells and the staining intensity. Staining intensity was scored as follows: 0, no staining; 1, weak staining; 2, moderate staining; and 3, strong staining. The score was defined as 0, 1, 2, 3 or 4 for 0, 1-10%, 11-50%, 51-80% and 81-100% of positively stained cells, respectively. The average of the lower and the higher staining intensities was calculated when an uneven distribution between staining intensity or multifocal immunoreactivity were observed. A final IHC score in the range 0-12 was obtained by multiplying the score by the staining intensity. IHC scores of 0, 1-4, 5-8 and 9-12 were considered as negative, weak, moderate and strong, respectively. Data were analyzed using Image-Pro Plus 6.0 software (Media Cybernetics, Inc.).

#### Western blotting

Proteins were extracted from the small intestine using RIPA lysis buffer (Beyotime Institute of Biotechnology) at 4˚C for 30 min. Protein concentration was determined using bicinchoninic acid protein assay kit. Proteins (50 µg) were separated by 10% SDS-PAGE and transferred onto a polyvinylidene fluoride membrane, which was subsequently blocked with 5% skim milk at room temperature for 1 h. Membranes were incubated at 4˚C overnight with the following primary antibodies: Mouse anti-F-actin (1:1,000; Abcam; cat. no. ab205); rabbit anti-claudin-2 (1:1,000; Zymed; Thermo Fisher Scientific, Inc.; cat. no. 516100); rabbit anti-Cdc42 (1:1,000; Abcam; cat. no. ab187643); rabbit anti-ZO-1 (1;1,000; Abcam; cat. no. ab96587); rabbit anti-occludin (1;1,000; Zymed; Thermo Fisher Scientific, Inc.; cat. no. 711500); and anti-GAPDH (1:1,000; Sigma-Aldrich: Merck KGaA; cat. no. G5262). Membranes were subsequently incubated with horseradish peroxidase (HRP)-conjugated Rb IgG (H+L; 1:3,000; OriGene Technologies, Inc.; cat. no. ZB-2301) or HRP-conjugated anti-mouse IgG (H+L; 1:3,000; OriGene Technologies, Inc.; cat. no. ZB-2305) at room temperature for 1 h. Bands were visualized using ECL (OriGene Technologies, Inc.; cat. no. sc-2048) and the relative expression of bands was normalized to the endogenous control GAPDH using Image-Pro plus 6.0 software (Media Cybernetics, Inc.).

#### Statistical analysis

SPSS version 24 (IBM Corp.) was used to analyze the data. Parametric analysis was performed as the data were normally distributed. Experiments were repeated independently at least three times, and the data were expressed as the mean ± standard deviation. χ^2^ test was used to analyze the differences between groups after converting categorical variables into percentages or frequencies. One-way ANOVA followed by Tukey's post hoc test was used to analyze differences among three or more groups. P<0.05 was considered to indicate a statistically significant difference.

## Results

### 

#### Mortality and BT rates

At 24 h following SAP induction, no death was observed in the SO and SO + carbachol group, whereas 30 rats died in the SAP group (mortality rate, 50%) and 18 rats died in the SAP + carbachol group (mortality rate, 30%). The results from bacterial species detection from the blood of rats in the SAP and SAP + carbachol groups are presented in Table I. No BT was observed in the SO and SO + carbachol group. However, numerous bacteria were detected in the blood of 18 rats in the SAP group (BT rate, 60%) and of 14 rats in the SAP + carbachol group (BT rate, 33.3%). In addition, the mortality (30% vs. 50%; P<0.05) and BT rates (33.3% vs. 60%; P<0.05) of rats in the SAP + carbachol group were significantly lower compared with rats in the SAP group.

#### Serum levels of lipase and amylase

**Figure 1:**
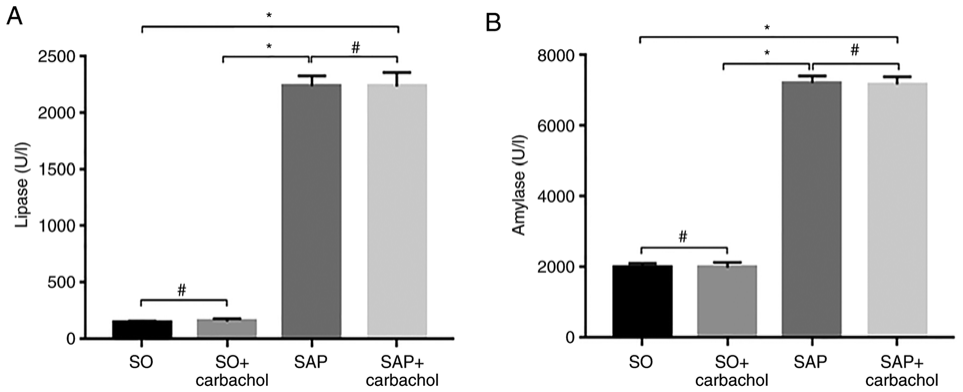
Serum levels of lipase and amylase in the blood of rats from the SO, SO + carbachol, SAP and SAP + carbachol groups. Levels of (A) lipase and (B) amylase were significantly higher in the SAP and SAP + carbachol groups compared with the SO. There was no difference between the SAP and SAP + carbachol groups, or between the SO and SO + carbachol groups. Results are presented as the means ± standard deviation (n=8 randomly selected mice in each group). ^*^P<0.05 and ^#^P>0.05. SAP, severe acute pancreatitis; SO, sham operation.

As presented in Fig. 1A, the serum levels of lipase in the SAP and SAP + carbachol groups were significantly higher compared with the SO group (P<0.05). In addition, there was no difference between the SAP and SAP + carbachol groups (P>0.05), and between the SO and SO + carbachol groups (P>0.05). These results were similar for the amylase serum level (Fig. 1B).

#### Intestinal and pancreatic histopathological scores

**Figure 2:**
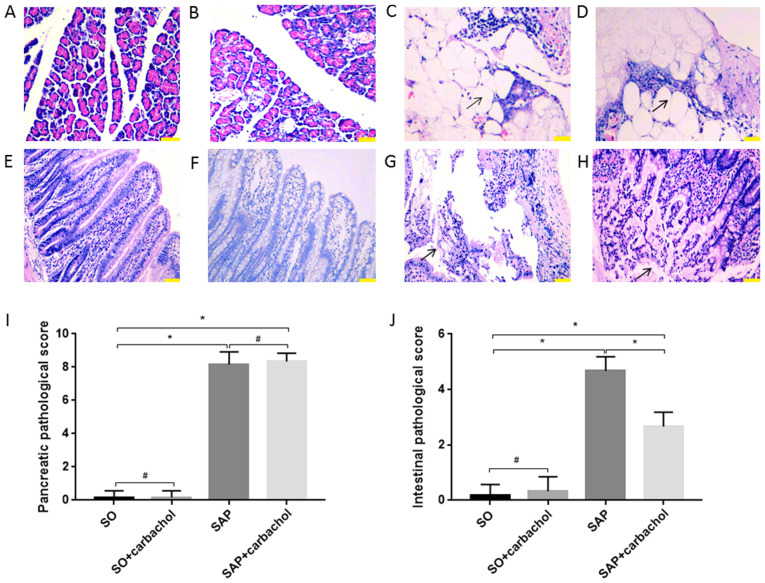
Representative sections of pancreatic and intestinal tissues stained with hematoxylin and eosin. Pancreatic tissue from the (A) SO and (B) SO + carbachol groups; no notable pathological changes were seen. Pancreatic tissue from the (C) SAP and (D) SAP + carbachol groups; hemorrhage and fat necrosis, interstitial edema, a fuzzy lobular structure, broad necrosis of acinar cells and infiltrating inflammatory cells were observed. Black arrows indicate adipocyte necrosis. Intestinal tissue of the (E) SO and (F) SO + carbachol groups; no significant pathological injury was observed. Intestinal tissue of the (G) SAP group; numerous inflammatory cells were observed to be infiltrating the thinning intestinal wall, as well as denuded villi with the lamina propria and dilated capillaries, and digestion and disintegration of the lamina propria. Black arrows indicate digestion and disintegration of the lamina propria. Intestinal tissue of the (H) SAP + carbachol group; mucosal glands reduced, tips denuded, local bleeding and necrosis, inflammatory cells infiltrated in the thinning intestinal wall. Black arrows indicate denuded tips of mucosal villi. Comparison of the pathological scores of (I) pancreatic and (J) intestinal tissues. Magnification, x200. Scale bar, 100 µm. Results are expressed as the mean ± standard deviation (n=6 randomly selected mice in each group). ^*^P<0.01 and ^#^P>0.05. SAP, severe acute pancreatitis; SO, sham operation.

The morphology of the intestinal mucosa and pancreas was evaluated. No injury was identified in the intestinal epithelium and pancreas of rats in the SO (Fig. 2A, E, I and J) and SO + carbachol (Fig. 2B and F-J) groups. Pancreatic and intestinal injury in the SAP and SAP + carbachol groups was significantly higher compared with SO group (P<0.01, Fig. 2A-D and I; P<0.01, Fig. 2E, G, H and J). Scores for pancreatic injury were not different between the SAP + carbachol and SAP groups (P>0.05; Fig. 2C, D and I); however, the score for intestinal injury in the SAP group was significantly higher compared with the SAP + carbachol group (P<0.01; Fig. 2G, H and J). Pancreatic tissue from SAP (Fig. 2C) and SAP + carbachol groups (Fig. 2D) exhibited hemorrhage and fat necrosis, interstitial edema, a disordered lobular structure, broad necrosis of acinar cells and infiltrating inflammatory cells. These results confirmed that the establishment of the SAP rat model was successful.

#### Expression of TJ proteins, F-actin and Cdc42 in intestine

**Figure 3:**
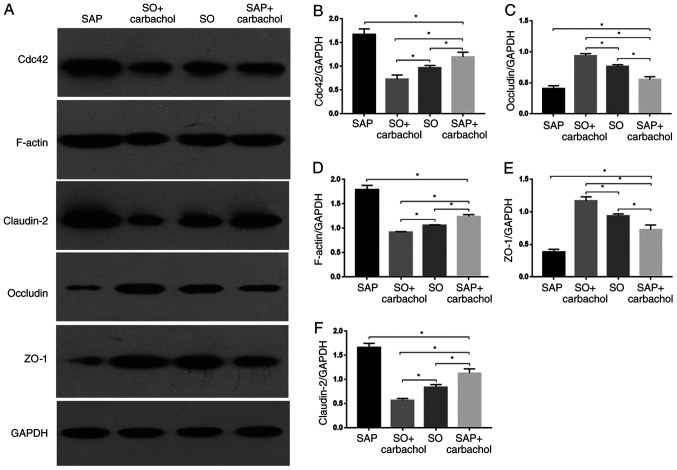
Western blotting analysis. Representative western blotting images of (A) claudin-2, occludin, ZO-1, F-actin and Cdc42 in the intestinal epithelium. Relative expression of (B) Cdc42, (C) occludin, (D) F-actin, (E) ZO-1 and (F) claudin-2 determined by optical densitometry. Results are expressed as the mean ± standard deviation (n=6 randomly selected mice in each group). ^*^P<0.05. SAP, severe acute pancreatitis; SO, sham operation; ZO-1, zonula occludens-1; Cdc42, cell division cycle 42.

The expression of Cdc42, F-actin and TJ proteins in the small intestine of rats in all groups was investigated by western blotting. Whereas claudin-2 expression was significantly increased (P<0.05; Fig. 3A and F), ZO-1 and occludin expression were significantly decreased in the SAP and SAP + carbachol groups (P<0.05; Fig. 3A, C and E) compared with the SO group. Furthermore, claudin-2 expression was significantly decreased (P<0.05; Fig. 3A and F), but ZO-1 and occludin expressions were significantly increased in the SO + carbachol group compared with the SAP and SAP + carbachol groups (P<0.05; Fig. 3A, C and E). Expression of ZO-1 and occludin was significantly increased in the SAP + carbachol group compared with the SAP group (P<0.05; Fig. 3A, C and E), whereas claudin-2 expression was significantly decreased in the SAP + carbachol group compared with the SAP group (P<0.05; Fig. 3A and F). In addition, Cdc42 and F-actin expression in the SAP and SAP + carbachol groups was significantly increased compared with the SO group (P<0.05; Fig. 3A, B and D); however, expression of these proteins was significantly decreased in the SO + carbachol group compared with the SO group (P<0.05; Fig. 3A, B and D). Expression of Cdc42 and F-actin was significantly decreased in the SAP + carbachol group compared with the SAP group (P<0.05; Fig. 3A, B and D).

#### Detection of Cdc42 by IHC

**Figure 4:**
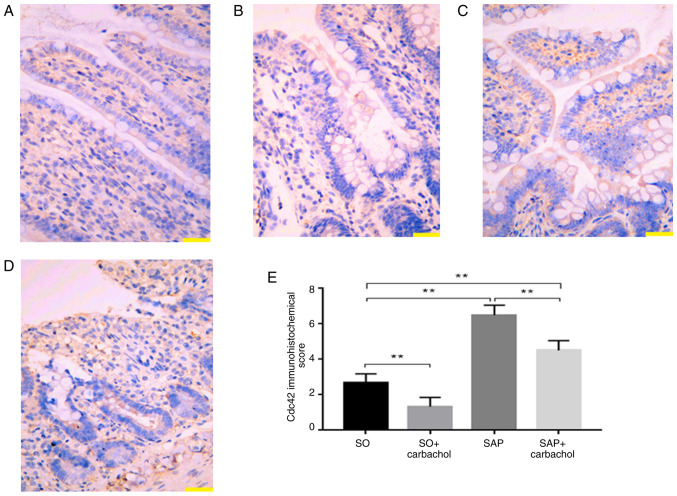
Representative images of Cdc42 expression by immunohistochemistry. Cytoplasmic staining (red) of small intestine in rats revealed a (A) low expression of Cdc42 in the SO group. (B) Low expression of Cdc42 in the SO + carbachol group (red). (C) High expression of Cdc42 in the SAP group (red). (D) Moderate expression of Cdc42 in the SAP + carbachol group (red). (E) Cdc42 expression was significantly increased in the SAP and SAP + carbachol groups when compared with the SO group. Furthermore, Cdc42 expression, in the SO + carbachol group was the lowest and the expression of Cdc42 was significantly decreased in the SAP + carbachol group compared with the SAP group. Magnification, x200. Scale bar, 100 µm. Results are expressed as the mean ± standard deviation (n=6 randomly selected mice in each group). ^**^P<0.01. SAP, severe acute pancreatitis; SO, sham operation; Cdc42, cell division cycle 42.

The detection of Cdc42 in rat intestinal epithelium was performed by IHC. As presented in Fig. 4, Cdc42 expression was significantly increased in the SAP and SAP + carbachol groups compared with the SO group (P<0.01; Fig. 4A and C-E). Furthermore, Cdc42 expression, in the SO + carbachol group was the lowest (P<0.01; Fig. 4B and E), and expression of Cdc42 was significantly decreased in the SAP + carbachol group compared with the SAP group, (P<0.01; Fig. 4C-E). These results were consistent with the results from the western blotting.

#### Detection of F-actin and TJ proteins by fluorescence microscopy

**Figure 5:**
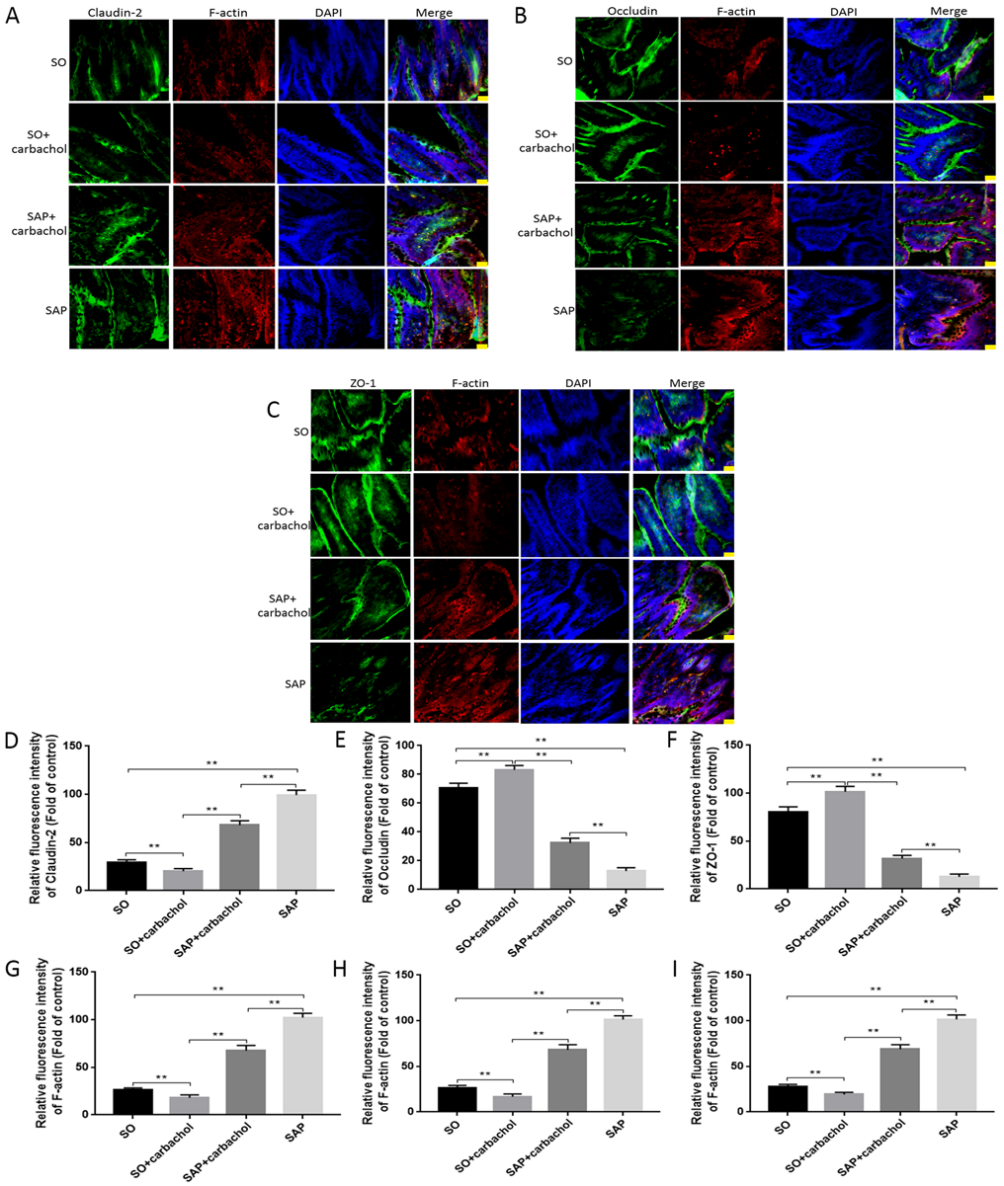
Immunofluorescent detection. Intestinal sections from rats in the SAP, SAP + carbachol, SO + carbachol and SO groups were triple stained with F-actin (red), DAPI (blue), and ZO-1 (green), occluding (green) or claudin-2 (green). (A) Claudin-2, F-actin and DAPI staining. (B) Occludin, F-actin and DAPI staining. (C) ZO-1, F-actin and DAPI staining. The fluorescence intensity of (D) claudin-2, (E) occludin and (F) ZO-1, and the respective F-actin fluorescence intensity from the staining experiments for (G) claudin-2, (H) occludin and (I) ZO-1, were analyzed. The results are expressed as the mean ± standard deviation (n=6 randomly selected mice in each group). Magnification, x200. Scale bar, 100 µm. ^**^P<0.01. SAP, severe acute pancreatitis; SO, sham operation; ZO-1, zonula occludens-1.

The expression of TJ proteins and F-actin in intestinal tissue, as well as nuclear staining, was detected by immunofluorescence triple staining. Claudin-2 (green), occluding (green) and ZO-1 (green) (Fig. 5A-C, respectively) were observed at the junction among intestinal epithelial cells and F-actin (red) was observed in the cytoplasm of intestinal epithelial cells. As presented in Fig. 5, occludin and ZO-1 staining intensity in the SAP + carbachol group was significantly higher compared with that in the SAP group (P<0.05; Fig. 5B, C, E and F). However, occludin and ZO-1 staining intensity was significantly higher in the SO + carbachol group compared with the SO group (P<0.05; Fig. 5B, C, E and F). Conversely, claudin-2 and F-actin staining intensity was significantly higher in the SAP group compared with the SAP + carbachol group (P<0.05; Fig. 5A, D and G), and significantly lower in the SO + carbachol group compared with the SAP + carbachol group (P<0.05; Fig. 5A, D and G). Combined with pathological results, the degree of intestinal damage was determined to be associated with the expression of TJ protein. These results were consistent with the results from the western blotting.

## Discussion

SAP is a critical condition characterized by the rapid failure of numerous organs and a high mortality rate (1). Infection of the pancreas and peripancreatic necrosis is the main cause of mortality in patients with late SAP (37).

Previous studies reported a significant correlation between the increased intestinal mucosal permeability and a poorer prognosis of SAP in patients (38,39). In cases of sepsis and multiple organ failure, there is a poor prognosis in surgical treatment of SAP (8,10). BT within the intestine is the main cause of systemic infection in patients with SAP. It was demonstrated that the main factor promoting BT is the destruction of the intestinal barrier (40-42). It is therefore crucial to protect the function of the intestinal barrier in patients with SAP in order to prevent and treat BT and subsequent systemic infection. The present study investigated the protective effect of carbachol on intestinal epithelial barrier function in rats with SAP and the underlying role of Cdc42/F-actin.

Carbachol is a cholinergic agonist that has been reported to serve a protective role in intestinal barrier dysfunction induced by LPS, TNF-α or following IBD (26,27). Octreotide is a synthetic somatostatin analogue, which inhibits glandular secretion. Previous studies reported that octreotide cannot significantly improve the incidence rate of complications and mortality associated with moderate to severe acute pancreatitis (43-45). Previous studies have revealed extensive necrosis of pancreatic acini or atrophy of pancreatic acini with a reduction of enzyme content in SAP (46,47). Li *et al* (29) therefore hypothesized that a large number of pancreatic acinar lesions and necrosis in patients with SAP lead to insufficient secretion from residual acinar tissue. At present, the protective effect of carbachol on intestinal barrier is clear. There was no significant difference in the severity of pancreatic injury between the SAP and SAP + carbachol treated groups. Furthermore, the severity of intestinal injury in the SAP + carbachol group was lower compared with that in the SAP group. Similarly, the BT and mortality rates in the SAP + carbachol group were lower compared with the SAP group. Serum levels of lipase and amylase in the SAP and SAP + carbachol groups were significantly higher compared with the SO group. In addition, no differences were identified in the serum levels of lipase and amylase between the SAP and SAP + carbachol groups, and between the SO and SO + carbachol groups. Further investigation using a larger sample size is required to validate these results. These findings suggested that carbachol may protect the function of the intestinal barrier in rats with SAP without aggravating pancreatic injury.

TJ proteins are transmembrane proteins localized between intestinal epithelial cells. These proteins are anchored by the peripheral membrane protein ZO-1 to cytoplasmic F-actin-based cytoskeleton proteins. This structure forms a physical barrier between intestinal epithelial cells, which is essential to maintain intestinal function (48,49). Cdc42 is a GTP-binding protein from the Rho family that regulates actin skeleton assembly and rearrangement (50,51). Proteins from this family have been demonstrated to regulate cell morphology, migration and endocytosis (52,53). The results from the present study confirmed the effect of Cdc42 on TJ proteins and F-actin. In the SAP + carbachol group, intestinal tissue injury was decreased, and the expression of ZO-1 and occludin was higher, whereas the expression of claudin-2, Cdc42 and F-actin was lower compared with the SAP group. BT rate was also lower in the SAP + carbachol group compared with the SAP group. These results indicated that ZO-1 and occludin may serve a crucial role in maintaining the intestinal barrier. However, this study demonstrated that claudin-2 was associated with intestinal barrier dysfunction, which was consistent with previous studies (19,30,54). The results of the current study confirmed that carbachol reduces the expression of Claudin-2, Cdc42 and F-actin, and increases the expression of ZO-1 and occludin in the small intestine of SAP rats. The results indicated that carbachol may protect the intestinal TJ barrier of rats with SAP by regulating the expression of Cdc42 and F-actin.

The present study had some limitations in terms of the evaluation of SAP-related intestinal injury and BT. A further study could collect ascites and detect GFP-*Escherichia coli* in an SAP rat model and discern whether ascites and bacteria translocation occurred in the abdominal cavity of rats. In this way, it would be possible to not only understand BT indirectly by 16S rRNA technology, but also to observe BT visually by microscopy. The results from the present study suggested that carbachol may serve a role in the protection of the intestinal barrier in SAP via Cdc42/F-actin; however the underlying mechanisms remain unknown.

In conclusion, to the best of our knowledge, the present study demonstrated for the first time that carbachol may protect the intestinal barrier in a rat model of SAP without aggravating pancreatic injury. Furthermore, the present study demonstrated the potential regulatory role of Cdc42/F-actin in the protection of the intestinal barrier function in SAP. Further investigation on the protective role of carbachol against intestinal barrier injury in SAP is essential to develop novel therapeutic strategies for patients with SAP.

## Figures and Tables

**Table I:** Bacteria species identified from the blood of rats in the SAP and SAP + carbachol groups.

A, SAP rats	
Rat number	Bacterial species
2	*Escherichia coli*
5	*Citrobacter freundii*
9	*Enterococcus aerogenes*
10	*Streptococcus pneumonia*
12	*Enterococcus faecium*
17	*Escherichia coli*
23	*Enterococcus aerogenes*
28	*Prevotella copri*
31	*Citrobacter freundii*
33	*Escherichia coli*
34	*Enterococcus faecium*
38	*Streptococcus pneumonia*
43	*Prevotella copri*
45	*Escherichia coli*
49	*Enterococcus aerogenes*
52	*Enterococcus aerogenes*
55	*Enterococcus faecium*
57	*Escherichia coli*
B, SAP + carbachol rats	
1	*Citrobacter freundii*
3	*Escherichia coli*
8	*Escherichia coli*
12	*Streptococcus pneumonia*
17	*Enterococcus aerogenes*
21	*Enterococcus faecium*
27	*Prevotella copri*
33	*Citrobacter freundii*
38	*Escherichia coli*
42	*Enterococcus faecium*
46	*Prevotella copri*
49	*Escherichia coli*
54	*Escherichia coli*
58	*Enterococcus aerogenes*

SAP, severe acute pancreatitis.

## Data Availability

The datasets used and/or analyzed during the current study are available from the corresponding author on reasonable request.
